# Networks Depicting the Fine-Scale Co-Occurrences of Fungi in Soil Horizons

**DOI:** 10.1371/journal.pone.0165987

**Published:** 2016-11-18

**Authors:** Hirokazu Toju, Osamu Kishida, Noboru Katayama, Kentaro Takagi

**Affiliations:** 1 Graduate School of Human and Environmental Studies, Kyoto University, Sakyo, Kyoto, Japan; 2 Tomakomai Experimental Forest, Field Science Center for Northern Biosphere, Hokkaido University, Aza-Takaoka, Tomakomai, Hokkaido, Japan; 3 Center for Ecological Research, Kyoto University, 2-chome, Hirano, Otsu, Shiga, Japan; 4 Teshio Experimental Forest, Field Science Center for Northern Biosphere, Hokkaido University, Aza-Toikanbetsu 131, Horonobe-cho, Teshio-gun, Hokkaido, Japan; University of Hyogo, JAPAN

## Abstract

Fungi in soil play pivotal roles in nutrient cycling, pest controls, and plant community succession in terrestrial ecosystems. Despite the ecosystem functions provided by soil fungi, our knowledge of the assembly processes of belowground fungi has been limited. In particular, we still have limited knowledge of how diverse functional groups of fungi interact with each other in facilitative and competitive ways in soil. Based on the high-throughput sequencing data of fungi in a cool-temperate forest in northern Japan, we analyzed how taxonomically and functionally diverse fungi showed correlated fine-scale distributions in soil. By uncovering pairs of fungi that frequently co-occurred in the same soil samples, networks depicting fine-scale co-occurrences of fungi were inferred at the O (organic matter) and A (surface soil) horizons. The results then led to the working hypothesis that mycorrhizal, endophytic, saprotrophic, and pathogenic fungi could form compartmentalized (modular) networks of facilitative, antagonistic, and/or competitive interactions in belowground ecosystems. Overall, this study provides a research basis for further understanding how interspecific interactions, along with sharing of niches among fungi, drive the dynamics of poorly explored biospheres in soil.

## Introduction

Fungi in soil constitute highly species-rich biological communities, playing pivotal functional roles in various types of terrestrial ecosystems [1–3]. In forest and grassland ecosystems, diverse mycorrhizal fungi promote the growth of host plants by providing soil nitrogen and phosphorus [4–6], while many host-specific pathogenic fungi attack plants [7], potentially promoting species coexistence in plant communities [8] (*sensu* [9]). Recent studies have also shown that plants ubiquitously interact with root-endophytic fungi [10–12], which may enhance host nutritional conditions and/or resistance to soil pathogens [13–15]. Moreover, saprotrophic fungi control the pace of nutrient cycles in both natural and agricultural ecosystems [3, 16] and entomopathogenic fungi (e.g., *Beauveria* spp.) inhibit the outbreaks of pest insects [17]. Thus, the knowledge of mechanisms organizing soil fungal communities is essential for managing agricultural ecosystems and restoring forests/grasslands. Due to the remarkable diversity of soil fungi [18], however, our understanding of soil fungal assembly processes has been limited.

Based on the emerging high-throughput sequencing technology [19, 20], recent studies have come to reveal the complex community structure of soil fungi in natural and agricultural ecosystems [21, 22]. Those studies have shown that community structure of soil fungi could be determined by various edaphic factors such as pH, nitrogen/phosphorus concentration, and tillage practices [21, 23] as well as dispersal limitation at both small and large spatial scales [24, 25]. Such researches based on high-throughput sequencing can further enhance our knowledge by estimating how biotic factors, i.e., interspecific interactions [26, 27], can organize patterns in fungal communities in the wild [12]. Positive (facilitative) and negative (competitive) interactions between species, in general, play pivotal roles in ecological community processes [28–30], but those interactions are often difficult to uncover especially in species-rich communities. Thus, although high-throughput sequencing is providing rich data for understanding the assembly processes of complex soil fungal communities [19, 20], we still have limited knowledge of the whole-community-scale patterns organized by the sharing of environmental preferences (niches) and positive/negative interspecific interactions.

In microbiological studies of human gut bacteria, however, researchers have tried to detect signs of potential niche sharing and interspecific interactions based on large high-throughput sequencing datasets [31–33]. Those microbiome studies focus on “co-occurrence” patterns of species across sequenced samples: i.e., pairs of species sharing niches and those in positive interactions are expected to co-occur more frequently than expected by chance in the same host (or environmental) samples [31–33]. These co-occurrence analyses have been applied also to community ecological analyses of fungi in plant root systems, highlighting importance of interspecific interactions in the fine-scale assembly processes of fungi [34, 35] (but see [36]). Furthermore, such community-scale analyses allow us to infer how diverse taxonomic/functional groups of fungi structure networks [37, 38] of potential interactions and how those networks are compartmentalized into “modules” [30, 39] of closely associated fungi [35]. Although pioneering studies have examined co-occurrence network structure of soil fungal communities [40, 41], it remains a major challenge to understand how functionally diverse fungi are grouped into those co-occurrence network modules.

In this study, we investigated the community-scale network structure of a soil fungal community based on high-throughput sequencing data of a cool-temperate forest in northern Japan. From each of the soil samples collected across a soil profile (9.8 m [length] × 1.0 m [depth]) in the forest, fungal community structure was revealed with Illumina sequencing. The community data were then used to infer the structure of a network depicting potential niche sharing and/or interspecific interactions at each sampling depth across the O (organic matter) and A (surface soil) horizons. We then analyzed how taxonomically and functionally diverse fungi constitute the networks of co-occurrences at fine spatial scales in soil. Specifically, the analysis allowed us to infer how mycorrhizal, endophytic, saprotrophic, and pathogenic fungi formed network modules of closely associated fungi. Thus, the analysis provided a basis for discussing, e.g., whether the fine-scale distribution of mycorrhizal fungi in soil could be correlated with or independent from that of non-mycorrhizal fungi. Overall, this study shows a way for detecting signs of possible niche sharing and/or interspecific interactions in complex communities in soil based on rich information provided by high-throughput sequencing.

## Materials and Methods

### Terminology

This study was designed to infer spatial niche differentiation and/or interspecific facilitative/competitive interactions in a soil fungal community. In general, DNA-barcoding-based analyses do not provide any direct evidences of niche differentiation or interspecific interactions [10, 42, 43]. Therefore, our aim was not revealing “common mycelial networks” linking fungal (and host plant) individuals/species [5, 44] but detecting sings of potential niche differentiation and interspecific interactions [42, 43]. Throughout this paper, we use the term “network” in a broad sense [38] irrespective of physical (mycelial) connections among fungi.

### Sampling and molecular analysis

The sampling was conducted in Teshio Experimental Forest, Hokkaido University, Japan (AKAGAWA 44.985950°N, 142.009036°E) on November 5, 2012: sampling permission was issued by Hokkaido University. The study site was located in a cool-temperate secondary forest, which consisted mainly of *Abies sachalinensis*, *Betula ermanii*, *Betula maximowiczii*, *Acer pictum*, and *Phellodendron amurense*. At the study site, we made a 1-m-deep soil profile along a 9.8-m horizontal line (S1 Fig) and sampled two replicate samples of 0.5 cm^3^ soil at each of 10 depth classes (2-cm above the boundary of the O and A horizons, the O-A boundary, and 3-cm, 5-cm, 10-cm, 15-cm, 20-cm, 30-cm, 50-cm, and 100-cm below the O-A boundary) with 20-cm horizontal intervals (i.e., 10 depths × 50 horizontal points = 500 sampling positions). The soil samples were stored at -25°C and then DNA extraction was conducted with a cetyltrimethylammonium bromide (CTAB) method [45].

For each of the 1000 samples (500 × 2 replicates), the internal transcribed spacer 1 (ITS1) region of fungi were PCR-amplified with the high-taxonomic-coverage primers ITS1-F_KYO1 and ITS2_KYO2 [46]. Each of the forward and reverse primers was fused with 3–6-mer Ns (for improving Illumina sequencing quality) [47] and a Illumina sequencing primer region (forward, 5’- TCG TCG GCA GCG TCA GAT GTG TAT AAG AGA CAG- [3–6-mer Ns]–[ITS1-F_KYO1] -3’; reverse, 5’- GTC TCG TGG GCT CGG AGA TGT GTA TAA GAG ACA G [3–6-mer Ns]—[ITS2_KYO2] -3’). The PCR reaction was conducted using the buffer and DNA polymerase system of Ampdirect Plus (Shimazu) with a temperature profile of 95°C for 10 min, followed by 37 cycles at 94°C for 30 s, 50°C for 60 s, 72°C for 60 s, and a final extension at 72°C for 7 min. The ramp rate was set to 1°C/sec to prevent the generation of chimeric amplicons [48]. P5/P7 Illumina adaptors were then added in the subsequent PCR using fusion primers with 8-mer index sequences for sample identification [49] (forward, 5’- AAT GAT ACG GCG ACC ACC GAG ATC TAC AC—[8-mer tag]—TCG TCG GCA GCG TC -3’; reverse, 5’- CAA GCA GAA GAC GGC ATA CGA GAT—[8-mer tag]—GTC TCG TGG GCT CGG -3’). The temperature profile was 95°C for 10 min, followed by 8 cycles at 94°C for 30 s, 55°C for 60 s, 72°C for 60 s, and a final extension at 72°C for 7 min (ramp rate = 1°C/sec). The PCR amplicons of the 1000 samples and 8 PCR negative control samples were pooled with equal volume after a purification/equalization process with AMPure XP Kit (Beckman Coulter). The pooled library was sequenced using the Illumina MiSeq sequencer of Graduate School of Human and Environmental Studies, Kyoto University (KYOTO-HE) (2 × 300 cycle sequencing kit) with 15% PhiX spike-in.

### Bioinformatics

As the MiSeq Reporter program does not remove sequencing reads with low quality values at index positions and it tolerates 1-base mismatches between input and output index sequences, default FASTQ files output by Illumina sequencers often contain “miss-indexed” sequencing reads. To prevent the potential demultiplexing errors, demultiplexing was conducted using the program Claident v0.2.2015.03.11 [50] after converting raw MiSeq BCL data into FASTQ data using the bcl2fastq v1.8.4 program distributed by Illumina. In the demultiplexing process, all the sequencing reads containing low quality (quality scores < 27) index sequences were eliminated and no mismatch between input and output index sequences was tolerated. The obtained forward and reverse sequencing reads were then fused with each other by the program PEAR v0.9.6 [51]. Among the 4,726,706 merged reads obtained, 36,563 were discarded because 10% or more of their nucleotides had low (< 27) quality values and/or because they were less than 150 bp in length (data deposition: DDBJ BioProject, PRJDB4971). Potentially chimeric reads were also eliminated with the program UCHIME v4.2 [52]. Noisy reads were removed as well in this process by the approach introduced previously [53] using Claident. The 3,862,747 reads that passed the filtering processes were clustered with a cutoff sequence similarity of 97% in a parallelized process of the Minimus for accurate assembling/clustering [54] as implemented in Claident and the obtained consensus sequences were then used as operational taxonomic units (OTUs) in the following statistical analyses. In the clustering process, reads of each sample were clustered beforehand with a 98% cutoff similarity: the results of the within-sample dereplication was used as guide information in order only to accelerate the 97% clustering process [35, 43]. OTUs whose sequencing reads were less than ten in all the samples were removed because their sequences could contain PCR/sequencing errors [55].

The remaining OTUs were then subjected to molecular taxonomic identification using the UCLUST consensus taxonomic assigner algorithm [56] with the UNITE ver.7 dynamic database [57] as implemented in QIIME [58]. However, our manual BLAST search of the identification results suggested that the fast but non-exhaustive database-search strategy of the UCLUST algorithm and the underrepresentation of soil fungal taxa in the UNITE database often resulted in erroneous identification at low taxonomic levels (e.g., family or genus levels).

Therefore, molecular taxonomic identification of OTUs was performed based on the database search algorithm of the query-centric auto-*k*-nearest-neighbor (QCauto) method [50] and subsequent taxonomic assignment with the lowest common ancestor (LCA) algorithm [59] using Claident [35, 43]. A benchmark analysis has shown that the QCauto-LCA pipeline allows the most accurate identification among the existing algorithms of automated taxonomic identification [50]. We applied the QCauto algorithm to the obtained OTUs using the databases provided by filtering out unreliable sequence entries from the NCBI “nt” database (downloaded from ftp://ftp.ncbi.nlm.nih.gov/ on January 27, 2015) [35, 43, 50]. The taxonomic identification results of both QCauto-LCA and UCLUST-UNITE approaches are shown in S1 Data.

Based on the molecular taxonomic identification results, non-fungal OTUs and 13 samples that contained possible laboratory contaminants were removed from the dataset. As two of the eight negative control samples contained fungal OTUs, the OTUs found from the two negative control samples were discarded (107 of 1887 OTUs). By combining sequencing reads of two replicate samples per sampling position, we obtained a sample (row) × fungal OTU (column) data matrix, in which a cell entry depicted the number of sequencing reads of an OTU at a sampling position. Presumably due to the presence of PCR inhibitors in soil samples, the number of obtained reads varied considerably among sampling positions (S1 Data). Therefore, the data matrix was rarefied to 1000 reads per sampling position (S2 Fig) using the “rrarefy” command of the vegan v2.2–3 package [60] of R v3.2.3 [61]. As a consequence, a matrix containing 1,221 OTUs from 303 sampling positions was obtained (S1 and S2 Data)). The functional group (guild) of the OTUs was inferred using FUNGuild v1.0 [62].

### Fungal OTU richness and community structure

We first examined how fungal diversity changed depending on the depth of sampling positions by calculating the mean number of fungal OTUs at each depth. Effects of sampling depth on fungal community structure were then evaluated by the permutational analysis of variance (PERMANOVA; 10,000 permutations) [63] using vegan. We also examined the homogeneity of dispersions over sampling depths with the permutational analysis for the multivariate homogeneity of dispersions (PERMDISP) [64]. Before the PERMANOVA and PERMDISP analysis, the *β*-diversity of the fungal compositions was calculated based on the Raup-Crick metric [65]. The differentiation of fungal community structure among sampling depths was inferred also with the nonmetric multidimensional scaling (NMDS). Because samples from 30-cm, 50-cm-, and 100-cm-deep positions included many outlier sampling positions in the NMDS ordination, data from these deepest three sampling depths were excluded from the NMDS visualization. We also analyzed how fungal community structure was spatially auto-correlated along the 9.80-m horizontal line based on a Mantel correlogram analysis using vegan (Raup-Crick *β*-diversity; 10,000 permutations).

To reveal patterns in the habitat differentiation of fungi across the soil profile in more detail, the vertical distribution of each fungal OTU was analyzed. Specifically, the habitat preference (*HP*) of a fungal OTU (*j*) for a sampling depth (*i*) was evaluated as follows:

$$HP(i,j)\;=\;[N_{\text{observed}}(i,j)\;–\text{ Mean }(N_{\text{ranodomized}}(i,j))]\;/\text{ SD }(N_{\text{ranodomized}}(i,j)),$$

where *N*_observed_ (*i*, *j*) was the number of samples from which a focal combination of a sampling depth and a fungus was observed in the original data, and the Mean (*N*_ranodomized_ (*i*, *j*)) and SD (*N*_ranodomized_ (*i*, *j*)) denoted the mean and standard deviation of the number of samples for a focal depth–fungus combination across randomized matrices. Randomized matrices were obtained by shuffling the depth-labels of the 303 samples in the data matrix (10,000 permutations). A larger positive *HP* value indicated a stronger preference of a fungus to a focal sampling depth, while a larger negative value represented stronger avoidance.

### Network structure

Using the fungal community data, we evaluated co-occurrences of soil fungi. In the community data matrix, more/less sequencing reads of a fungal OTU can be observed in the sampling points in which another OTU has more reads. To reveal such sings of potential positive/negative interactions in each pair of fungal OTUs, we used two statistical methods: the sparse correlations for compositional data (SparCC) method [31] and the sparse inverse covariance estimation for ecological association inference (Spiec-Easi) method [32]. The former method relies on correlations between species in compositional data matrices [31] and the latter uses sparse neighborhood and inverse covariance selection algorithms [32]. In a previous study comparing statistical methods for inferring co-occurrence networks, the SparCC and Siec-Easi methods and an additional method returned qualitatively consistent results [35]. For each sampling depth, an input matrix was prepared by selecting fungal OTUs that appeared in 1/3 or more sampling positions at each depth. As the use of rarefied matrices (i.e., matrices in which the total number of sequencing reads per sample is equalized) can introduce artifacts in the co-occurrence analyses [32], read-count data before rarefaction was used (S1 Data). Sampling depths below 3 cm were not examined in this analysis due to the small number of sampling positions from which enough number of sequencing reads were obtained (S1 Data). In the SparCC analysis, the cutoff value of absolute correlation coefficients was set to 0.4. In the Spiec-Easi analysis, the Meinshausen and Bühlmann (MB) algorithm [66] was applied. For each sampling depth, networks of potential positive and negative associations were drawn based respectively on the results of the SparCC and Spiec-Easi analyses using the igraph v.1.0.1 package [67] of R. The Fruchterman-Reingold algorithm [68] was used for the layout of fungal OTUs in the networks. Although various statistical tools for detecting network modules have been available, as previously applied to a co-occurrence network analysis of root-associated fungi [35], the network structure revealed in this study was too fragmented to be subjected to statistical modularity analyses. Therefore, we focused on how multiple functional groups of fungi constituted discrete clusters in the network of each sampling depth.

## Results

### Fungal OTU richness and community structure

**Fig 1:**
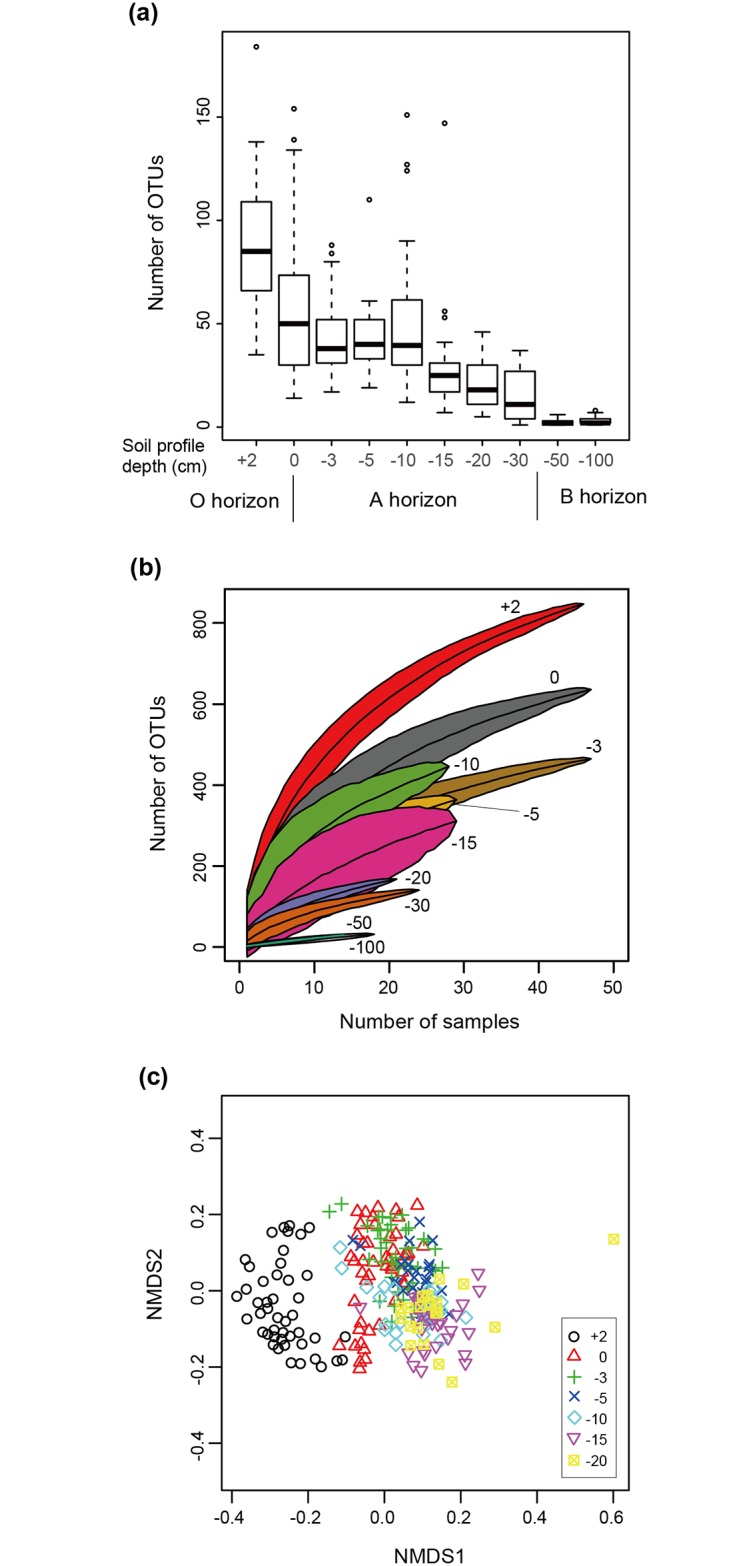
Variation in the diversity and community structure of soil fungi along sampling depth. (a) Number of fungal OTUs per sampling position. A box indicates the first and third quartiles and a thick line shows the median of a focal sampling depth. (b) Relationship between the number of sampling positions and that of fungal OTUs at each sampling depth. The number of sampling positions from which 1000 or more sequencing reads were obtained decreased along sampling depth. (c) NMDS visualization of fungal community structure.

The mean number of fungal OTUs per sample significantly decreased in deeper sampling positions (*F*_1,8_ = 11.2, *P* = 0.01; Fig 1a). Although the number of sampling positions from which 1000 or more sequencing reads were obtained decreased at deeper positions, the total number of fungal OTUs observed was saturated with a smaller number of horizontal sampling positions in deeper vertical positions (Fig 1b). The PerMANOVA analysis suggested that the fungal community structure varied among sampling depths (df = 1, *F*_model_ = 47.7, *P* < 0.0001; Fig 1c). The subsequent PERMDISP analysis further indicated that the observed differentiation of community structure was attributed, at least partly, to the heterogeneity of dispersions among sampling depths (df = 9, *F* = 21.0, *P* < 0.0001). Spatial autocorrelations among horizontal sampling points disappeared within 2 m in the sampling site (S3 Fig).

**Fig 2:**
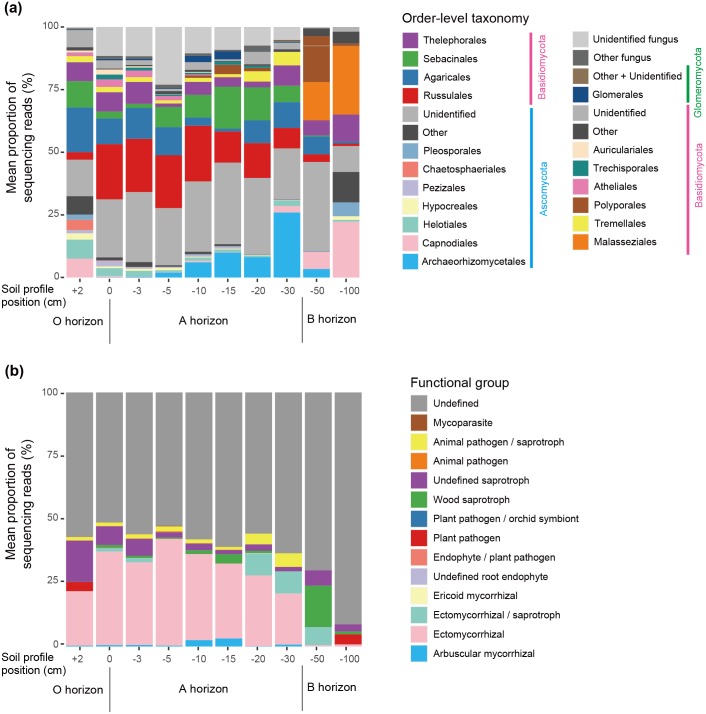
Variation in fungal community compositions across sampling depths. (a) Taxonomic composition at the order level. (b) Compositions of fungal functional group (guild) inferred by FUNGuild.

The taxonomic composition of detected fungi is shown in Fig 2a. The samples from the O horizon, which consisted of organic matters, had higher proportions of Capnodiales, Helotiales, Hypocreales, and Chaetosphaeriales fungi than those from the A horizon. In contrast, Archaeorhizomycetales fungi were found mainly from samples at the lower part of the A horizon (10-30-cm-deep samples). Among Basidiomycete orders encompassing ectomycorrhizal fungi, Russulales were found mainly from the A horizon, while Agaricales, Sebacinales, and Thelephorales appeared at both O and A horizons. The fungal community of the B horizon (subsurface layer reflecting physical and chemical properties of parental material) was characterized by *Malassezia* OTUs (Malasseziales), which are commonly detected from animal skins but are reported also from forest soils and plant roots (e.g., [69]). Regarding functional group (guild), the O and A horizons were dominated by ectomycorrhizal fungi (Fig 2b) as expected by the dominance of ectomycorrhizal plants (*Abies* and *Betula*) in the forest.

**Fig 3:**
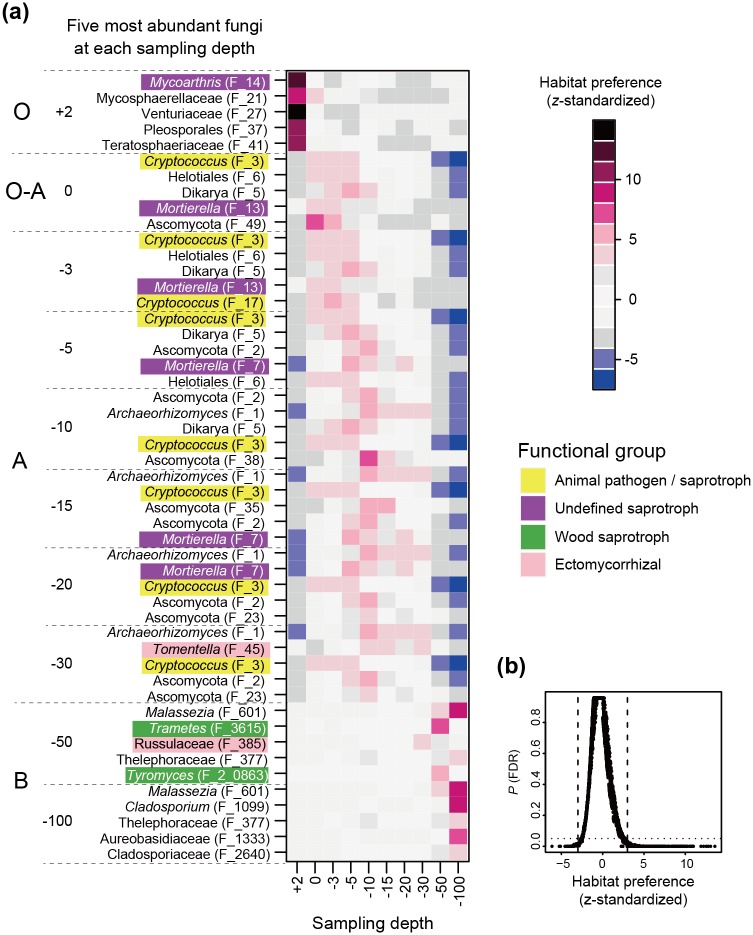
Habitat preferences of soil fungi. (a) Habitat preferences of top-5 fungi with the largest sample counts at each sampling depth. The ID of each fungal OTU (F_xx) corresponds to that in S1 Data. (b) Relationship between *z*-standardized habitat preferences and the *P* values (false discovery rate) obtained from a randomization analysis.

The five most commonly observed fungal OTUs at each sampling depth had strong signs of preferences for soil horizons (Fig 3a). Basically, fungal OTUs whose *HP* scores exceeded 3 displayed statistically significant preferences for certain sampling depth(s) (Fig 3b). The fungi that dominated the O horizon had very strong preferences for their habitat, while the fungi frequently observed from the A horizon displayed weaker but statistically significant preferences for the soil horizon (Fig 3*a*). The fungi that dominated the B horizon (e.g., *Malassezia* sp. [F_601; Malasseziales] and *Cladosporium* sp. [F_1099; Capnodiales]) showed strong preferences for the horizon, forming a fungal community distinct from that of upper horizons (Fig 3a).

### Network structure

**Fig 4:**
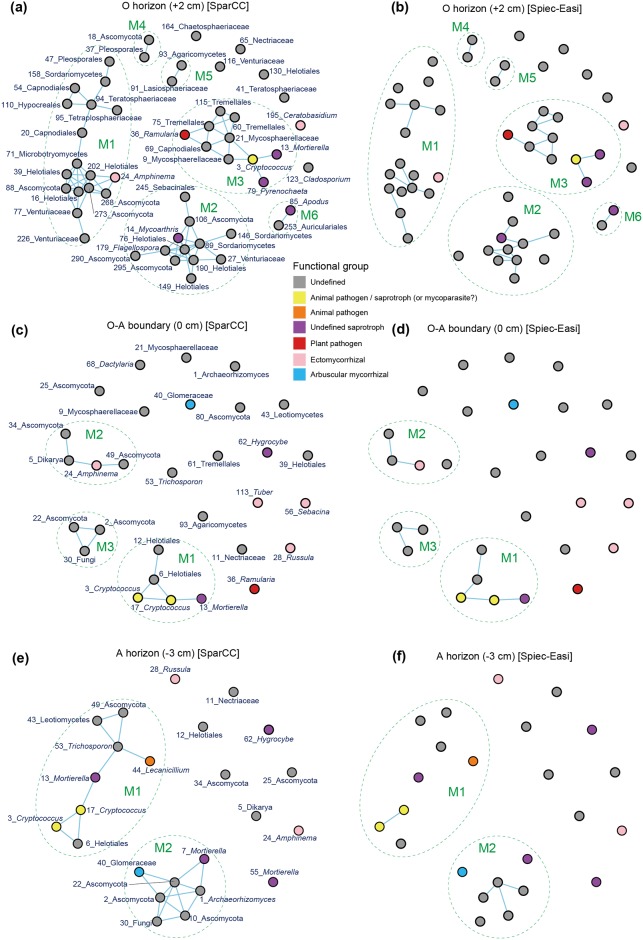
Positive co-occurrence networks. Based on the SparCC (left) and Spiec-Easi (right) methods, pairs of fungi that co-occurred frequently in the same soil samples were indicated. Fungal OTUs for which neither positive nor negative (Fig 5) interactions with other OTUs were inferred do not appear in the networks. Discrete network modules (clusters) are indicated by dotted lines. The ID and the lowest taxonomic information are shown for each fungal OTU. (a-b) O horizon (+2 cm). (c-d) O-A boundary (0 cm). (e-f) A horizon (-3 cm).

The co-occurrence network of each sampling depth was compartmentalized into some discrete modules (clusters) of closely associated fungi, which were expected to share environmental preferences (niches) and/or interact with each other in positive (facilitative) ways (Fig 4). Between the two statistical methods applied, the Spiec-Easi method returned more conservative results as discussed previously [32]: i.e., the modules inferred in the SparCC analysis were further compartmentalized in the Spiec-Easi analysis (Fig 4). Hereafter, we define modules as fungal OTU sub-communities with two or more positive co-occurrence links in the SparCC analysis.

Partly due to the differences in fungal community structure (Figs 2 and 3), the compositions of fungi forming network modules differed among sampling depths. For example, both the SparCC and Spiec-Easi analyses inferred positive associations between an ectomycorrhizal fungus in the genus *Amphinema* (F_24) and unidentified Ascomycota and Dikarya fungi (F_5 and F_34) at the O-A boundary, while such modules involving ectomycorrhizal fungi were not observed at the 3-cm-deep positions (Fig 4). Instead, a module including an arbuscular mycorrhizal fungus was inferred with the SparCC analysis (but not with the Spiec-Easi analysis) at the A horizon (3-cm-deep) (Fig 4).

Although many of the fungi analyzed in this study were unable to be identified at the genus or family levels, the detected network modules involved fungal OTUs belonging to various functional guilds. For example, fungi in the order Helotiales, which encompassed diverse fungi interacting with plants as endophytes and decomposing dead plant materials [70], were frequently observed in the modules observed in our data. Some of those Helotiales fungi (e.g., F_202 in module 1 at the O horizon and F_6 in module 1 at the A horizon) co-occurred with ectomycorrhizal, pathogenic, and/or saprotrophic fungi at each sampling depth (Fig 4). There were some modules including fungi in the genus *Cryptococcus*, whose teleomorphs (fungi in the genus *Filobasidiella*), were known as parasites of entomopathogenic fungi in the genus *Lecanicillium* [71, 72]. Indeed, *Cryptococcus* (= *Filobasidiella*) (F_3 and F_7) and *Lecanicillium* (F_44) fungi were detected in the same module at the A horizon (3-cm-deep) in the SparCC analysis (Fig 4).

**Fig 5:**
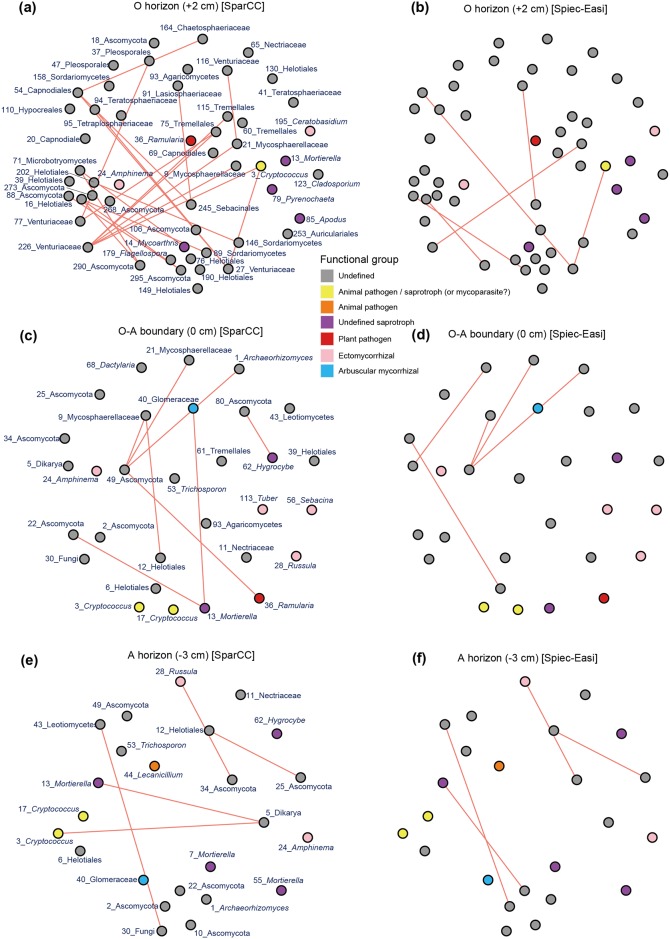
Negative co-occurrence networks. Based on the SparCC (left) and Spiec-Easi (right) methods, pairs of fungi displaying segregated distributions across the soil samples were indicated. The ID and the lowest taxonomic information are shown for each fungal OTU. (a-b) O horizon (+2 cm). (c-d) O-A boundary (0 cm). (e-f) A horizon (-3 cm).

The SparCC and Spiec-Easi analyses also suggested potential negative interactions between fungal OTUs (Fig 5). An ectomycorrhizal fungus in the genus *Russula* (F_28), for example, displayed patterns negatively correlated with those of an unidentified Ascomycota fungus (F_34) at the A horizon (3-cm-deep). Likewise, an arbuscular mycorrhizal fungus (F_40) had a network link of potential negative interactions with a *Mortierella* fungus (F_13) at the O-A boundary in the SparCC analysis.

## Discussion

We analyzed herein how taxonomically and functionally diverse fungi form networks of fine-scale co-occurrences in soil. Although saprotrophic, mycorrhizal, endophytic, and pathogenic fungi are often analyzed separately in studies of belowground fungi (but see [21]), they can share niches and positively/negatively interact with each other in natural and agricultural ecosystems. Such a viewpoint of correlated (and interdependent) ecological processes of functionally diverse fungi is expected to enhance our understanding of the “whole ecosystem functions” governed by soil microbial communities [73]. Ecosystem management programs optimized for the use of mycorrhizal fungi, for example, may increase/decrease entomopathogenic fungi through microbial interaction networks, resulting in the suppression/outbreaks of pest insects. Thus, in establishing frameworks for predicting whole fungal community dynamics in soil, the combination of high-throughput sequencing and co-occurrence network analyses provides an invaluable research basis.

Although our data included fungi without detailed taxonomic and natural history information, the detected network modules represented characteristic associations among different functional groups of fungi (Fig 4). The presence of modules consisting of mycorrhizal fungi and taxonomically diverse soil-inhabiting fungi suggests that the status of plant–fungus mycorrhizal symbioses can be influenced by external biotic interactions in soil and vice versa. While previous studies on root-associated fungal communities have suggested facilitative interactions between mycorrhizal and endophytic fungi within host root systems [34, 35], this study illuminates the potential impacts of poorly explored soil fungi on mycorrhizal symbioses. Ecologically intriguing patterns were observed also in the modules that did not involve mycorrhizal fungi. Potentially myco-parasitic fungi in the genus *Filobasidiella* (*= Cryptococcus*), for instance, co-occurred with its potential host fungus in the genus *Lecanicillium* [72], which was known as an entomopathogenic taxon [71]. This finding suggests that positive co-occurrence patterns do not necessarily represent facilitative or mutualistic interactions but also antagonistic (i.e., exploiter–victim) interactions. In contrast to such possible antagonistic interactions shown in positive co-occurrence networks, mutually exclusive (competitive) interactions are expected to appear in the networks of negative co-occurrences (segregated patterns). The negative co-occurrence networks included potential competitive interactions between poorly investigated soil-inhabiting fungi and those involving mycorrhizal fungi (Fig 5).

The co-occurrence analysis allows us to raise working hypotheses on interactions among different functional groups of fungi, but the observed patterns may be explained, at least partly, by sharing of niches in soil environments [74] (but see [75]). One possible way for evaluating potential effects of niche sharing is to use new lines of statistical methods, which divide effects of direct interspecific interactions from those of shared environmental preferences [76–78]. Although these methods have been used to analyze data collected in observational community ecological studies, our preliminary analysis on human gut microbiome datasets [79] has shown that the new statistical approach is applicable to high-throughput sequencing-based datasets if data of physical/chemical environmental conditions are available (Toju et al., in review). However, because this study was designed to reveal fine-scale co-occurrences of soil fungi, we did not have enough volume of samples for the measurements of soil environmental conditions (e.g., pH and nitrogen concentrations). Therefore, evaluating the relative contributions of niche sharing and direct interspecific interactions remains important challenges for understanding mechanisms organizing soil fungal communities.

As expected by the vertical gradient in OTU richness (Fig 1) and taxonomic compositions (Figs 2 and 3), the complexity of co-occurrence networks varied across the O and A soil horizons (Figs 4 and 5). However, given the number of sampling positions analyzed at each sampling depth (≤ 50), the number of network links might be underestimated even at the O horizon. In general, small number of samples can lead to pseudo-negative results in the estimation of potential positive/negative co-occurrence patterns [34]. Therefore, discrete modules (clusters) observed in the present analysis may be recognized as parts of larger modules when we increase the number of samples in future studies. It should be also taken into account that our present analysis did not detect negative co-occurrence links between mycorrhizal fungi despite the fact that strong competitive interactions between mycorrhizal fungi have been reported repeatedly in studies of root-associated fungi [26, 27, 80]. In addition to sample size, the choice of methods for co-occurrence analyses is expected to affect statistical results. Although a benchmark test has reported that the Spiec-Easi method, which depends on algorithms for sparse neighborhood and inverse covariance selection, performs better than other methods used in human microbiome studies (e.g., SparCC) [32], it would be productive to find patterns (network links) consistent among results based on multiple statistical methods [35].

In ecology and mycology, high-throughput sequencing has become a standard tool for community ecological analyses, but utmost care is required when designing molecular experimental protocols and interpreting results. Specifically, because DNA barcoding analyses do not provide any direct information of the natural history or life cycles of detected fungi, complementary microscopic observations and experimental investigations are awaited for further understanding niche differentiation and interspecific interactions in soil fungal communities. In addition, continual efforts to improve the molecular experimental and bioinformatic procedures are necessary. When sequencing-read counts are used as quantitative information representing relative abundance of OTUs, compositional bias resulting from molecular experimental steps may influence community data analyses [81]. In PCR, for example, index sequences in fusion primers could cause template-sequence-specific bias of amplification across samples [82]. Thus, in this study, the PCR amplification of template DNA was conducted with PCR primers without index sequences and, subsequently, index sequences were added to the amplicons in the 2nd PCR process with a small number of cycles in order to avoid such potential PCR bias. Taxonomic coverage of PCR primers and the gene copy number of marker regions can also introduce bias to read-count data [81], but their influence to SparCC and Spiec-Easi analyses may be limited if consistent experimental conditions were applied to all samples throughout the PCR and sequencing procedures.

If continuous attention is paid to potential pitfalls in molecular experimental and statistical procedures, the combination of high-throughput sequencing and co-occurrence network analyses will provide new options for investigating complex community dynamics in nature. Although ecological inferences of this study is based entirely on the spatially restricted sampling in a cool-temperate forest, the molecular experimental and statistical methods can be applied to any soil microbial communities. Knowledge of microbe–microbe co-occurrence networks will help us design new lines of studies for further understanding mechanisms organizing species-rich communities. By sequencing the genomes of multiple fungal species in the same network modules, for example, we may be able to examine how those frequently co-occurring fungi have complementary sets of functional genes. Another important extension of the network theoretical approach is to analyze potential facilitative/competitive interactions among different taxa of organisms. That is, by obtaining the community compositional data of not only fungi but also bacteria in a high-throughput sequencing run, we can infer how the two groups of soil microbes interact with each other in natural and agricultural ecosystems. Further studies based on high-throughput sequencing and co-occurrence analyses will help us explore poorly investigated biosphere in soil.

## Supporting Information

S1 Data Fungal community matrix and taxonomic information. (XLSX)

S2 Data ITS1 sequences of fungal OTUs. (FASTA)

S1 Fig Photographs of the study site. (a) Making of the 9.80-m trench. (b) Entire picture of the 9.80-m trench. (c) Enlarged view of the soil profile.
(TIF)

S2 Fig Relationship between the number of sequencing reads and that of fungal OTUs. (TIF)

S3 Fig Mantel’s correlogram analysis of the spatial auto-correlation of fungal community structure. Distance classes with significant Mantel’s correlation indices (*r*) are indicated by filled squares.
(TIF)
