# Supplementary figures and images for: Networks Depicting the Fine-Scale Co-Occurrences of Fungi in Soil Horizons
Source: PLoS One. 2016 Nov 18;11(11):e0165987. doi: 10.1371/journal.pone.0165987 (PMC5115672; doi:10.1371/journal.pone.0165987)

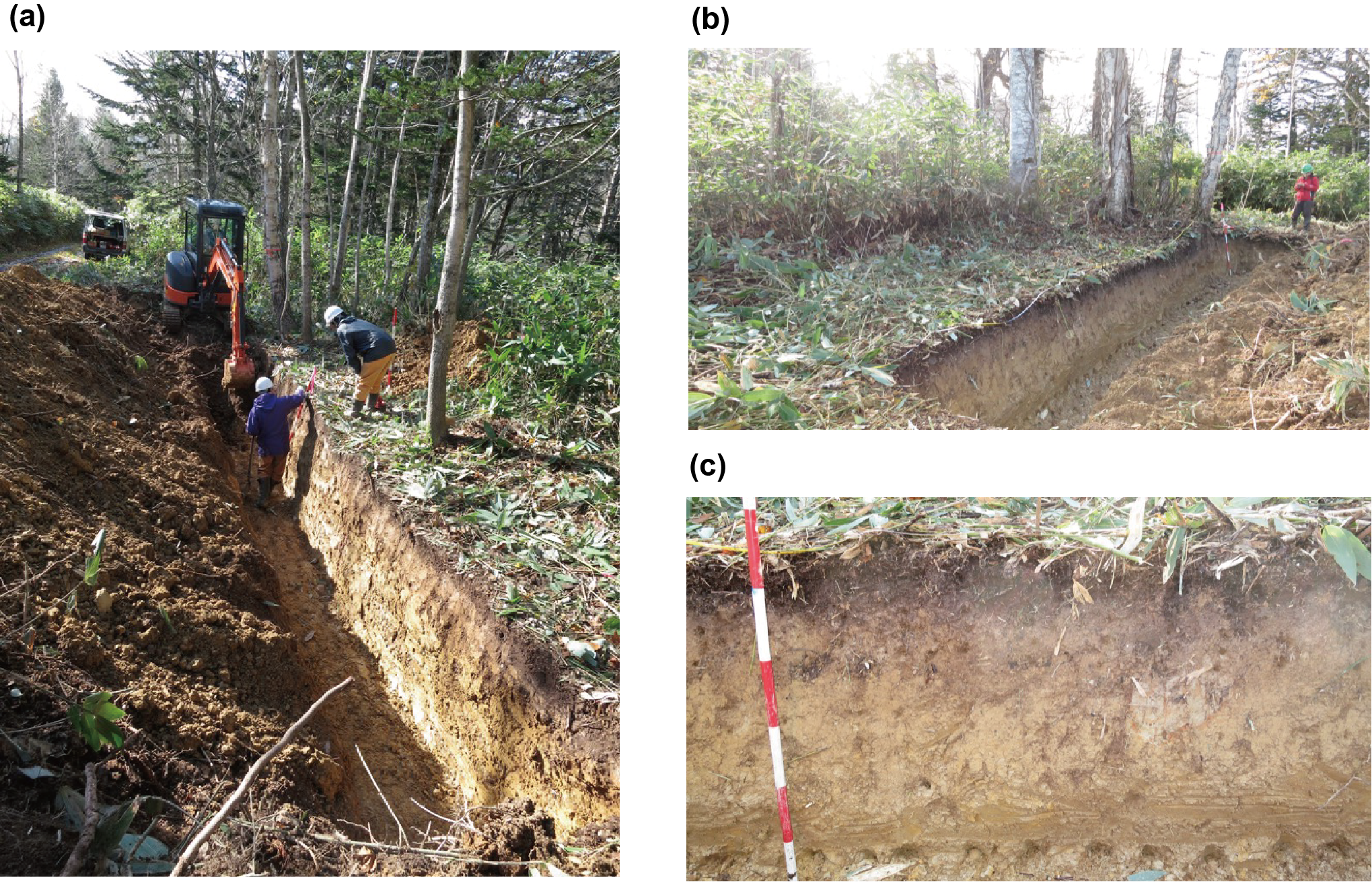

Supplement: S1 Fig — (a) Making of the 9.80-m trench. (b) Entire picture of the 9.80-m trench. (c) Enlarged view of the soil profile. (TIF) [file pone.0165987.s003.tif]

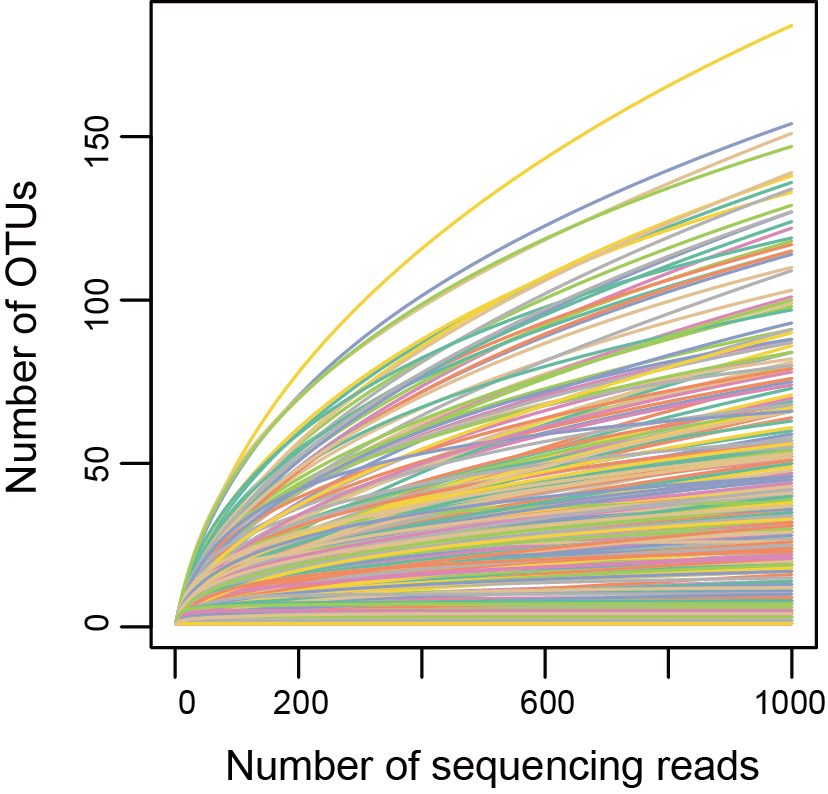

Supplement: S2 Fig — (TIF) [file pone.0165987.s004.tif]

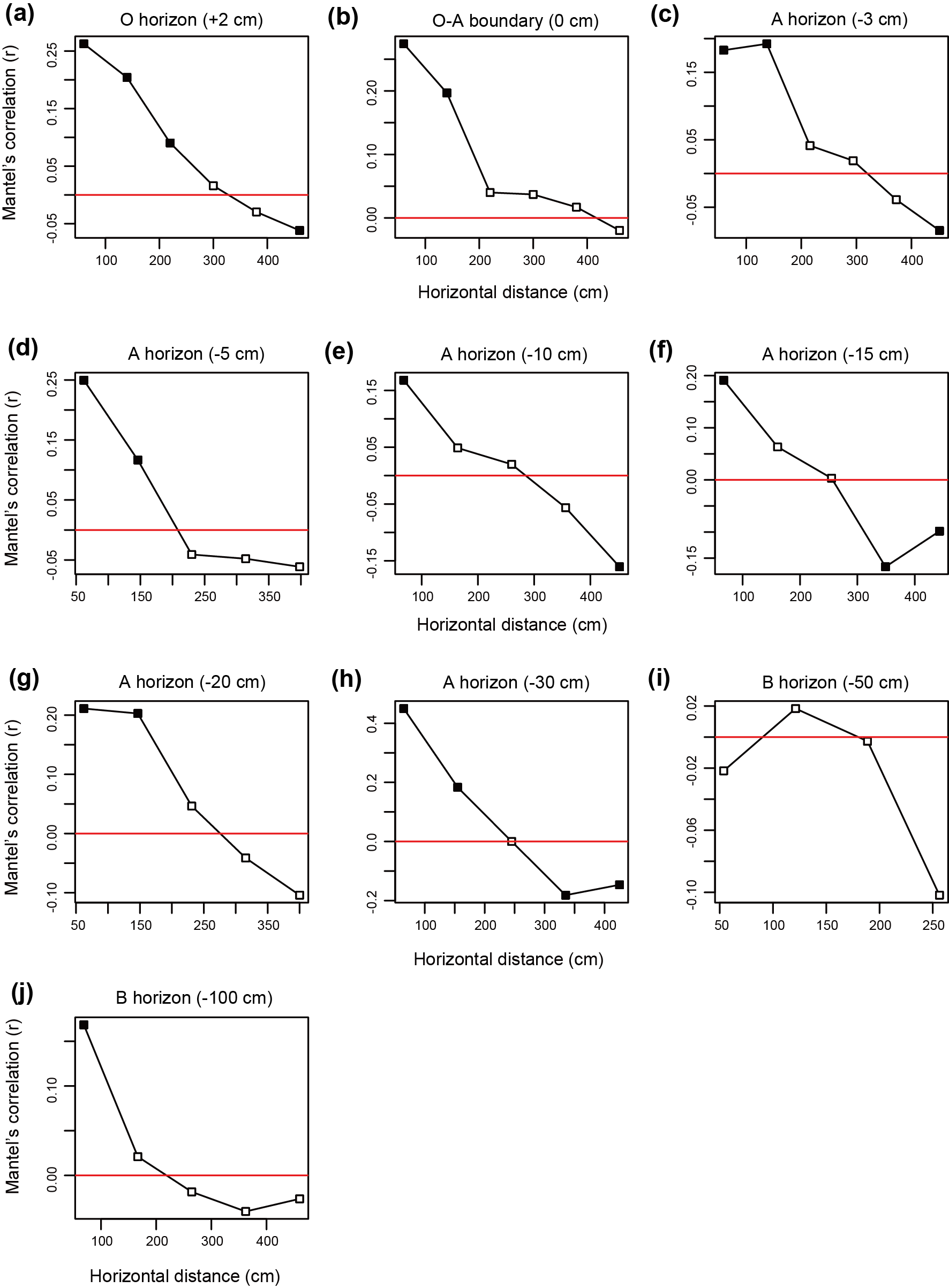

Supplement: S3 Fig — Distance classes with significant Mantel’s correlation indices (r) are indicated by filled squares. (TIF) [file pone.0165987.s005.tif]
